# Prediction of Spectral Parameters in Er^3+^, Dy^3+^ and Nd^3+^ Doped Oxide Glasses via cGAN-Enhanced Hybrid Modeling

**DOI:** 10.3390/s26113296

**Published:** 2026-05-22

**Authors:** Liumiao Xie, Hengxin Yang, Xiangfu Wang

**Affiliations:** 1 College of Electronic and Optical Engineering & College of Flexible Electronics (Future Technology), Nanjing University of Posts and Telecommunications, Nanjing 210023, China; 2 Anhui Province Key Laboratory of Environment-Friendly Polymer Materials, Anhui University, Hefei 230601, China

**Keywords:** rare-earth-doped glasses, Judd–Ofelt parameters, machine learning, conditional GAN, spectral prediction

## Abstract

The Judd–Ofelt (J–O) intensity parameters and oscillator strengths are key to understanding the optical transition properties of rare-earth-doped glasses. However, the scarcity of experimental samples and the complex nonlinear relationship between composition and spectral properties pose significant challenges to accurate predictions. To address this, we propose a generalizable framework that integrates conditional generative adversarial network (cGAN)-based data augmentation with an attention-embedded artificial neural network (ANN)–support vector regression (SVR) hybrid model. The cGAN generates physically plausible virtual samples to enrich data distribution and enhance generalization in sparse compositional regions. The attention mechanism in the ANN identifies critical compositional features, which are then leveraged by SVR for robust regression of parameter trends. The framework demonstrates high predictive accuracy for Er^3+^-doped glasses, achieving R^2^ values above 0.93 for Ω_2_, Ω_4_, and Ω_6_, and exhibits strong generalization performance on independent Dy^3+^- and Nd^3+^-doped datasets without task-specific retraining, confirming its practical applicability across multiple rare-earth ions. The model maintains consistency across diverse glass host systems (tellurite, borate, phosphate, silicate/germanate, heavy-metal oxide), and the attention analysis reveals feature importance aligned with established glass chemistry principles. Demonstrated on Er^3+^, Dy^3+^, and Nd^3+^, with potential for a broader range of rare-earth ions through transfer learning and future dataset extensions, this approach offers a data-driven, physics-informed tool for the targeted design of rare-earth optical materials in next-generation optical sensors.

## 1. Introduction

Rare-earth-doped oxide glasses, with their high solubility for rare-earth ions, tunable refractive indices, and excellent forming properties, have become promising materials for 1.5 μm fiber amplifiers, broadband solid-state lasers, mid-infrared emission, bioimaging, and advanced optical sensors, potentially replacing traditional crystalline and fluoride materials in some cases [1,2]. Studies have shown that the optical transition probabilities, spectral linewidth, and laser emission cross-sections of these materials are significantly influenced by the glass composition, local structure, and hydroxyl content. Among these, the Judd–Ofelt intensity parameters (Ω_2_, Ω_4_, Ω_6_) and their derived properties, such as oscillator strengths and radiative lifetimes, are key factors for understanding and optimizing their performance in various photonic applications, particularly in fluorescence intensity ratio (FIR)-based temperature sensors, rare-earth-doped fiber-optic point sensors, and optical bio-probes [3,4,5,6].

The traditional research process typically involves a workflow of “composition selection → glass melting → absorption/emission spectroscopy → J–O fitting analysis,” which relies heavily on trial-and-error experimentation. This approach struggles to uncover the complex nonlinear relationships between the glass composition and spectral properties, especially across broad compositional spaces. While machine learning and neural network approaches have been applied to predict Ω_2_, Ω_4_, and Ω_6_ [7,8,9,10,11,12], most of these studies focus on single glass systems and fail to provide effective cross-system predictions. A review of current research published between 2021 and 2026 highlights a growing trend of applying advanced algorithmic models to predict these parameters. Recent advances include ensemble learning models such as XGBoost and LightGBM optimized with Gray Wolf Optimization for rare-earth-doped phosphate glasses [10], random forest regression for Dy^3+^-doped glasses [11], deep learning models for tellurite glasses [12], and statistical learning for Er^3+^-doped tellurite glasses [7]. Despite these intensive recent efforts, most contemporary studies focus on isolated glass systems and lack sufficient capacity for small-sample data augmentation, which severely limits their use in the high-throughput design of rare-earth-doped glasses across broad compositional spaces.

In recent years, generative adversarial networks (GANs) have shown great potential in materials science, with successful applications in data augmentation, materials discovery, and property prediction [13,14,15,16]. Motivated by this, we propose a GAN-based framework for data augmentation and spectral parameter modeling to overcome the limitations of traditional methods. This framework allows for high-accuracy, cross-composition predictions of spectral parameters in multi-system rare-earth-doped glasses, even under small-sample conditions. This work not only enhances our understanding of the intrinsic relationships between the glass composition, structure, and optical absorption properties but also provides a valuable tool for the targeted design of efficient optical materials for sensor applications.

## 2. Judd–Ofelt Theory

The 4f–4f transitions of rare-earth ions are electric-dipole forbidden in free ions but become allowed in solid crystal fields due to mixing with higher-energy states. The Judd–Ofelt theory provides a quantitative framework for describing the intensity of such electric-dipole transitions [17,18]. Experimental oscillator strengths are obtained from absorption spectra and compared with the calculated values to fit the J–O parameters Ω_2_, Ω_4_, and Ω_6_ [19].

In recent years, significant progress has been made in refining and expanding the applications of the Judd–Ofelt theory. Recent advancements have introduced robust computational frameworks, local-field corrections, and rigorous theoretical adaptations that improve the accuracy of spectral predictions [20,21]. These developments, including improved automated fitting algorithms and comprehensive error evaluations, provide a much more reliable foundation for calculating transition probabilities and assessing the optical properties of rare-earth-doped materials [22,23].

### 2.1. Experimental Oscillator Strength

For a transition from the ground state to an excited state, the experimental oscillator strength is derived from the absorption spectrum as:

(1) $$f_{\exp}=\frac{m_{e}c^{2}}{\pi e^{2}N}\int \varepsilon (\nu )d\nu$$

where me is the electron mass, *c* is the speed of light, *e* is the electron charge, *N* is the ion concentration, ε(ν) is the absorption coefficient, and *ν* is the wavenumber. The integration covers the effective absorption band of the transition, reflecting the overall interaction strength between the electronic transition and the external optical field.

### 2.2. Calculated Oscillator Strength

The calculated electric-dipole oscillator strength is expressed as:

(2) $$f_{\mathrm{ED}}=\frac{8\pi^{2}m_{e}c\nu }{3h\left(2J+1\right)}\frac{\left(n^{2}+2{)}^{2}\right.}{9n}\sum_{\lambda =2,4,6}\Omega_{\lambda }{\left|\langle S^{\prime },L^{\prime },J^{\prime }\left\|U^{\left(\lambda \right)}\right\|S,L,J\rangle \right|}^{2}$$

where *h* is Planck’s constant, *ν* is the transition wavenumber, *n* is the refractive index, and ${\left|\langle \left\|U^{\left(\lambda \right)}\right\|\rangle \right|}^{2}$ are the squared reduced matrix elements. This expression highlights the direct dependence of oscillator strength on line strength and transition energy, encapsulating the intrinsic connection between the electronic distribution and the optical transition probability.

### 2.3. Judd–Ofelt Parameter Expression

The electric-dipole line strength is expanded as:

(3) $$S_{\mathrm{ed}}=\sum_{\lambda =2,4,6}\Omega_{\lambda }{\left|\langle S^{\prime },L^{\prime },J^{\prime }\left\|U^{\left(\lambda \right)}\right\|S,L,J\rangle \right|}^{2}$$

where the reduced matrix elements are computed from the atomic theory and tabulated in the literature [20]. The parameters Ω_2_, Ω_4_, and Ω_6_ reflect the local coordination symmetry, bonding character, and host polarizability, respectively. A least-squares fitting of the experimental and calculated oscillator strengths yields the J–O parameters.

### 2.4. Magnetic-Dipole (MD) Contribution

Although the Judd–Ofelt (J–O) theory primarily focuses on forced electric-dipole (ED) transitions, magnetic-dipole (MD) contributions must be explicitly included for transitions obeying the selection rules ΔJ = 0, ±1 (excluding 0 ↔ 0). The MD oscillator strength is given by

(4) $$f_{MD}=\frac{8\pi^{2}mc\nu }{3h(2J+1)}\;n^{3}\;{\left|\langle \psi J\left\|L+2S\right\|\psi^{\prime }J^{\prime }\rangle \right|}^{2}$$

where the squared matrix element of the magnetic-dipole operator L+2S is obtained from intermediate-coupling calculations and tabulated in the literature [24]. The local-field correction factor for MD transitions is $\chi_{MD}=n^{3}$.

The total calculated oscillator strength then becomes

(5) $$f_{exp}=f_{ED}+f_{MD}$$

where $f_{ED}$ is the electric-dipole term given in Equation (2) and the MD term uses the expression above.

In Er^3+^-doped glasses, the lowest-energy transition ^4^I_15/2_ → ^4^I_13/2_ has a non-negligible MD component that contributes approximately 15% to the total oscillator strength and line strength [25,26]. This transition is one of the most commonly included in our compiled dataset. Neglecting the MD contribution would introduce a systematic bias, particularly in the fitted Ω_6_ parameter, and increase the root-mean-square deviation of the least-squares fitting procedure. Therefore, all oscillator strengths used in this work incorporate both ED and MD terms exactly as reported in the original publications [27,28,29,30,31,32,33,34,35,36,37,38,39,40,41,42,43,44,45,46].

Errors originating from the Judd–Ofelt least-squares fitting procedure can be significant and must be properly accounted for. As discussed in the foundational work of Walsh [19] and detailed in the recently developed open-source LOMS.cz computational platform [47], the root-mean-square (RMS) deviation between the experimental and calculated oscillator strengths, together with the uncertainties in the fitted parameters (ΔΩ_2_, ΔΩ_4_, ΔΩ_6_), should be systematically reported to evaluate the reliability of the obtained Judd–Ofelt parameters. In the present study, we adopt the Judd–Ofelt parameters and oscillator strengths exactly as reported in the original literature sources [27,28,29,30,31,32,33,34,35,36,37,38,39,40,41,42,43,44,45,46], all of which were obtained through standard least-squares fitting procedures that already incorporate both electric-dipole and magnetic-dipole contributions. Future extensions of this dataset will employ the LOMS.cz platform for automated uncertainty quantification and combinatorial Judd–Ofelt recalculation, thereby further enhancing consistency and reproducibility across multi-source literature data.

Note that any J-mixing contributions present in the experimental data are inherently absorbed into the fitted Ω_λ_ values reported in the original literature. The machine learning model therefore learns composition–property trends that already encapsulate these higher-order effects, and explicit J-mixing correction is not required for the trend-learning scope of this work.

## 3. Materials and Methods

### 3.1. Dataset and Feature Construction

Data were rigorously compiled from the published literature on Er^3+^-doped oxide-based glasses [27,28,29,30,31,32,33,34,35,36,37,38,39,40,41,42,43,44,45,46]. This compilation represents a comprehensive and exhaustive extraction of currently available, high-quality open-literature data that report full compositional details alongside experimental and calculated oscillator strengths. This process yielded a dataset where each sample includes both well-defined input features and output variables. The input features consist of a 24-dimensional glass composition vector and a glass system category label: based on a clustering analysis and chemical composition characteristics, all samples were classified into four categories, namely tellurite, borate–Li, phosphate, and heavy-metal oxide, which were encoded as integers and further subjected to one-hot encoding during the modeling process. The output variables include the Judd–Ofelt parameters (Ω_2_, Ω_4_, Ω_6_) as well as the experimental and calculated oscillator strengths corresponding to multiple characteristic transitions. All Judd–Ofelt parameters and oscillator strengths in the compiled dataset were taken directly from the original publications [27,28,29,30,31,32,33,34,35,36,37,38,39,40,41,42,43,44,45,46] and are used with full awareness of potential fitting uncertainties, as discussed in detail in Section 2.4 and Ref. [47].

The literature-derived Judd–Ofelt parameters used in this work follow the procedures reported in each original publication. While this enables large-scale data-driven modeling, the absolute comparability across different studies is inherently limited without standardized combinatorial Judd–Ofelt (C-JO) recalculation, as emphasized in recent work [24]. The present study therefore focuses on learning relative composition–property trends that are robust across minor methodological variations, including differences in selected intra-4f transitions, magnetic-dipole inclusion/exclusion, and band-assignment choices.

When addressing potential discrepancies in the band assignment reported in specific studies, including the work of Gaafar et al. [27], the illustrative samples in Table 1 and Table 2 draw exclusively on the final reported Judd–Ofelt parameters and oscillator strengths, rather than the raw assignments of absorption bands. We acknowledge, however, that some values taken from the literature, including those reported in Ref. [27], may carry minor inconsistencies in the band assignment, as highlighted in prior work [25]. These entries were retained for two critical reasons: the model delivers excellent predictive accuracy on the independent test set, with R^2^ values above 0.93 for all Judd–Ofelt parameters, and it demonstrates strong generalization performance when applied to an independent Dy^3+^ and Nd^3+^ dataset. These results collectively confirm that the dominant composition–property trends are reliably captured. Moving forward, expansions of this dataset will leverage the combinatorial approach and the LOMS.cz platform [47] to recalculate all parameters sourced from the literature, a measure that will further enhance the consistency and reproducibility of the full dataset.

To ensure the fundamental reliability of the dataset despite its stemming from a multi-source literature, we conducted a strict curation of the reported fitting quality. Across the compiled Er^3+^-doped samples, the reported root-mean-square (RMS) deviations for the Judd–Ofelt fitting procedure typically fall well within the acceptable range of 1 × 10^−6^ to 5 × 10^−6^, confirming that the foundational data quality of the original publications is statistically sound. Furthermore, the variance introduced by differing band-assignment methodologies is mitigated by a highly overlapping set of intra-4f transitions used across the sources. Specifically, the hypersensitive transition ^4^I_15/2_ → ^2^H_11/2_ and the ^4^I_15/2_ → ^4^I_13/2_ transition are almost universally utilized across our curated dataset. This high degree of transitional consensus provides a robust, comparable baseline for the machine learning algorithms.

Inputs were standardized using the training set mean and standard deviation:

(6) $$x_{\mathrm{std}}=\frac{x-\mu }{\sigma }$$

Outputs were similarly standardized to ensure balanced optimization across multiple targets.

The final real experimental dataset comprised 67 samples, with the system-wise distribution being: tellurite (49), borate–Li (7), phosphate (4), and heavy-metal oxide (6). Approximately 300 cGAN-generated samples per system were added, yielding a total augmented dataset of ~1267 samples for training and validation. The dataset was randomly split into training, validation, and test sets in an approximate 7:1.5:1.5 ratio, with the generated samples included only the in training set.

For illustration, selected tellurite glass samples (S1–S5) with their composition are presented in Table 1, and their corresponding spectral parameters in Table 2 [27].

As shown in Table 2, five distinct absorption peaks were utilized for fitting the Judd–Ofelt parameters in these representative samples. The rationale for utilizing this specific subset of peaks is that these transitions are highly pronounced, reliably measurable, and free from severe overlapping with adjacent bands, which is critical for minimizing the least-squares fitting uncertainties. Furthermore, to ensure the statistical reliability of the extracted parameters, the error evaluation—specifically the root-mean-square (RMS) deviation between the experimental and calculated oscillator strengths—was carried out following the rigorous evaluation methodology detailed in the recent literature [23].

Two separate validation datasets were established for the universality evaluation. One dataset contained approximately 50 Dy^3+^-doped glass samples, covering tellurite glass, borate glass, silicate and germanate glass, and heavy metal oxide glass systems [9]. The other dataset consisted of 87 Nd^3+^-doped glass samples, including tellurite glass, borate glass, phosphate glass, and silicate and germanate glass [48,49,50,51,52,53,54,55,56,57,58,59,60,61,62,63]. The same data preprocessing procedures were adopted for both datasets to verify the generalization performance across different rare earth ions.

### 3.2. cGAN Architecture and Training

The generator in our model is based on the Wasserstein GAN with Gradient Penalty (WGAN-GP) framework. It maps 32-dimensional Gaussian noise vector and a condition vector to synthetic samples. The target vector is constructed by concatenating the glass composition features (number of components determined by the input dataset) with the Judd–Ofelt parameter Ω_2_. The system category is represented as a one-hot encoded vector, with its dimensionality equal to the number of unique system clusters present in the training data.

The generator consists of a four-layer fully connected network with hidden dimensions of 256. Each hidden layer is followed by batch normalization and a LeakyReLU activation function (slope 0.2), and the final output layer uses a Tanh activation to match the range of the MinMax-scaled data (−1 to 1). The discriminator also employs a multi-layer perceptron with a hidden dimension of 256, but it splits the forward pass to compute a critic score for the WGAN loss and to extract intermediate features for a feature matching loss.

Training follows the WGAN-GP procedure. The discriminator is updated five times per generator update. The loss function for the critic includes a gradient penalty term (coefficient λ = 10) to enforce the 1-Lipschitz constraint. The generator is optimized using a combination of adversarial loss and a feature matching loss (coefficient λ = 10) which minimizes the L2 distance between the real and fake features extracted by the discriminator. The models are trained for 5000 epochs with a batch size of 32 using the Adam optimizer (β_1_ = 0.5, β_2_ = 0.9).

After generation, the synthetic samples are inverse-transformed from the [−1, 1] scaling back to their original ranges. To ensure physical validity, the composition components are clipped to be non-negative and renormalized such that their sum equals 100 mol%. The generated Ω_2_ values are clipped to lie within the minimum and maximum bounds observed in the real dataset.

To complete the set of optical parameters, a separate multi-output artificial neural network is trained to map composition and Ω_2_ to the remaining Judd–Ofelt parameters (Ω_4_ and Ω_6_) and oscillator strengths. This network is a three-layer perceptron with LeakyReLU activations and a hidden dimension of 64, trained using the mean squared error loss. The predictions from this network, when applied to the cGAN-generated samples, ensure that all derived parameters maintain physical consistency with the input composition and the hypersensitive Ω_2_ parameter.

### 3.3. Hybrid Multi-Parameter Prediction Model

The model consists of two main components: an attention-enhanced multi-output artificial neural network (ANN) and separate RBF-SVR models for each output variable.

The input includes a composition vector and a one-hot encoded system vector whose dimension equals the number of unique system_cluster categories in the dataset. An attention module, implemented as a two-layer MLP with sigmoid activation, computes the feature weights. These weighted features are passed through a backbone with two hidden layers: the first hidden layer has 256 units, and the second produces a 64-dimensional embedding. Both hidden layers use LeakyReLU activation. The embedding is then projected linearly to generate the output targets, which correspond to the columns listed in TARGET_COLS.

After generating the embeddings from the best validation model, each output target is refined using an independent RBF-SVR model trained on the combined training and validation embeddings. The SVR hyperparameters are set manually (C = 10.0, ε = 0.01) and the kernel coefficient γ is chosen automatically via the scikit-learn “scale” strategy.

### 3.4. Model Training and Evaluation

Real samples were split into a 7:1.5:1.5 ratio (train/val/test). A validation-based early stopping was applied to the ANN. The final evaluation used strictly the test split, reporting R^2^ and the MAE for both ANN and hybrid (SVR-on-embedding) predictions.

(7) $$R^{2}=1-\frac{\sum_{i}(y_{i}-{\hat{y}}_{i}{)}^{2}}{\sum_{i}(y_{i}-\bar{y}{)}^{2}},\;\;\;\;\;\;\;\;\;\mathrm{MAE}=\frac{1}{N}\sum_{i}\left|y_{i}-{\hat{y}}_{i}\right|$$

## 4. Results and Discussion

### 4.1. Distribution Consistency and Physical Plausibility of cGAN-Generated Data

**Figure 1:**
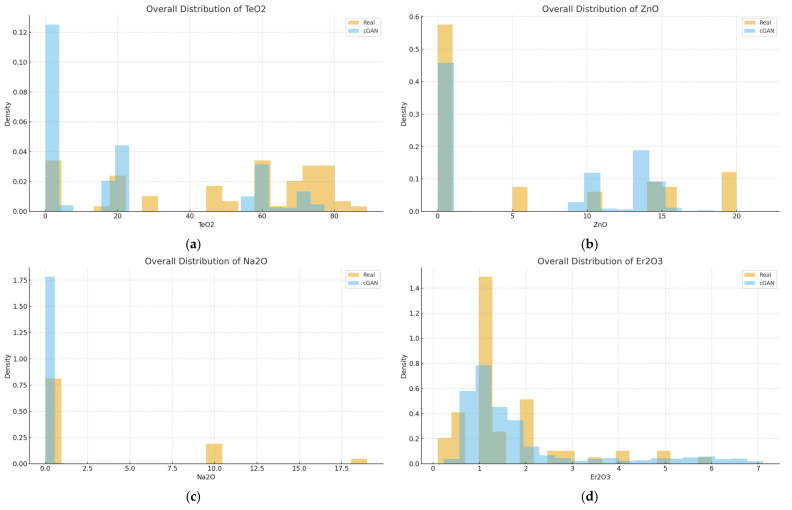
Comparison of one-dimensional distributions of key components and Ω_2_ for real and cGAN-generated glass data: (**a**) TeO_2;_ (**b**) ZnO; (**c**) Na_2_O; (**d**) Er_2_O_3_.

**Figure 2:**
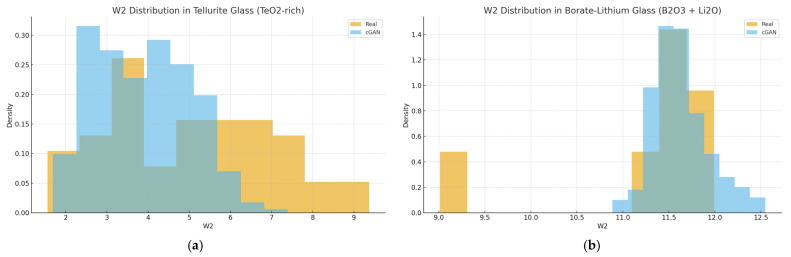

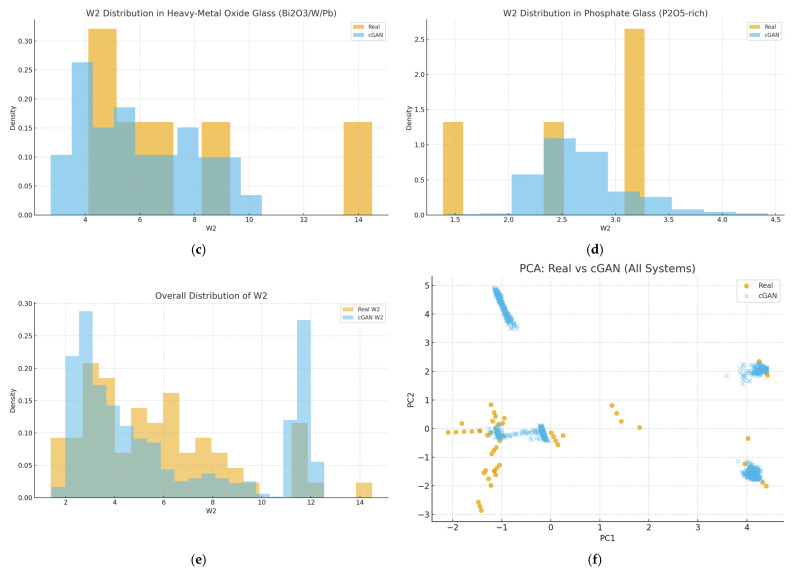
System-resolved Ω_2_ distributions and PCA cluster structure for real and cGAN-generated glass systems: (**a**) tellurite glass, (**b**) borate-lithium glass, (**c**) heavy-metal oxide glass, (**d**) phosphate glass, (**e**) overall Ω_2_ distribution, (**f**) PCA visualization. (Note: The labels “W2” in the subfigures denote the Judd–Ofelt parameter Ω_2_, with units of 10^−20^ cm^2^).

The cGAN-generated samples closely match the real data distribution. One-dimensional marginal distributions (Figure 1) and system-resolved Ω_2_ distributions (Figure 2a–d) show nearly identical overlaps between the real and generated samples for both major network formers and the hypersensitive Ω_2_ parameter. Principal component analysis (PCA) projections (Figure 2f) confirm that the generated samples occupy the same low-dimensional space as the real samples, maintaining distinct clusters across the glass systems.

The quantitative validation through the two-sample Kolmogorov–Smirnov (KS) tests (Table 3) shows high *p*-values (e.g., *p* = 0.07 for Ω_2_) and low KS statistics, indicating no significant statistical difference between the real and generated distributions. These results, along with the strict post-generation constraints—such as non-negative compositions, renormalization to 100 mol %, and Ω_2_ clipping to the observed ranges—confirm that the cGAN produces physically realistic virtual samples. This high-fidelity augmentation is essential for addressing the skewed real dataset and enhancing the model generalization to sparse composition regions without introducing unphysical artifacts.

### 4.2. Simulated Spectral Database and Oscillator Strength Consistency

**Figure 3:**
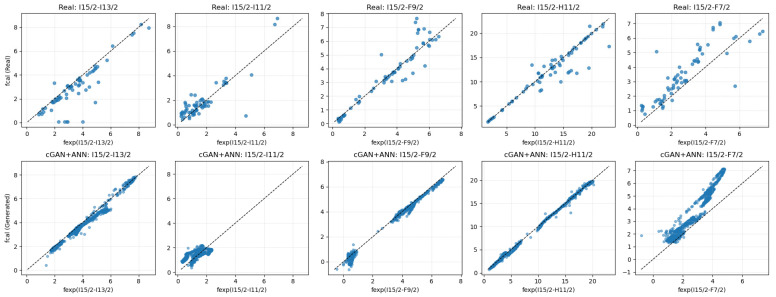
Scatter plot of *f*_exp_ and *f*_cal_ for each transition for real and cGAN-generated glass data.

Figure 3 shows a scatter plot comparing the experimentally measured oscillator strengths with the cGAN-predicted values for each transition. The plot demonstrates a strong correlation between the real and generated data, with the cGAN successfully preserving the physical correlations observed in the real samples. Notably, the generated data maintains realistic error characteristics, especially for the high-energy transitions.

This consistency in the oscillator strengths confirms that the cGAN generates physically plausible samples and accurately replicates the error distributions found in the experimental data. These results highlight the model’s effectiveness in extending spectral databases and ensuring reliable predictions in situations with limited experimental data.

### 4.3. Predictive Performance on Er^3+^-Doped Glasses

**Figure 4:**
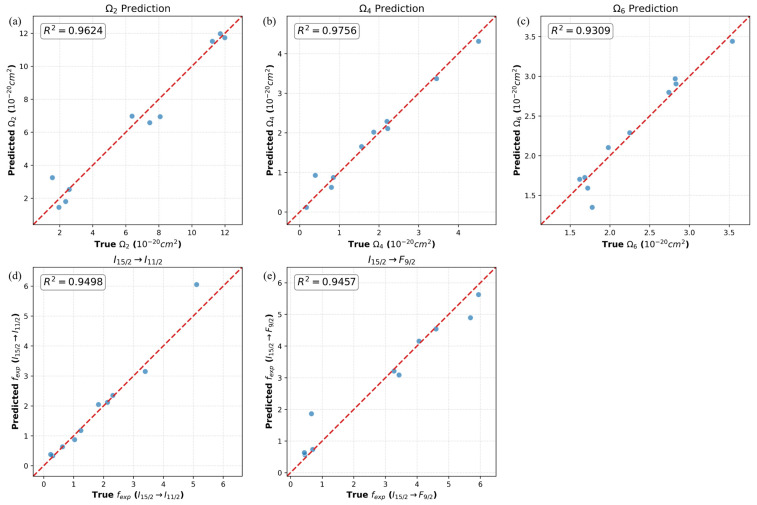
Scatter plots comparing experimental and predicted values for Judd–Ofelt parameters and selected experimental oscillator strengths in Er^3+^-doped glasses. (**a**) Ω_2_. (**b**) Ω_4_. (**c**) Ω_6_. (**d**) I_15/2_ → I_11/2_. (**e**) I_15/2_ → F_9/2_.

The attention-embedded ANN–SVR hybrid model demonstrates superior predictive performance on the independent test set for Er^3+^-doped glasses. Specifically, it achieves coefficients of determination (R^2^) exceeding 0.93 for all Judd–Ofelt parameters: R^2^ = 0.9629 for Ω_2_, R^2^ = 0.9756 for Ω_4_, and R^2^ = 0.9309 for Ω_6_ (Table 4, Figure 4). Additionally, the model accurately predicts the selected experimental oscillator strengths across various transitions, with R^2^ values ranging from 0.8322 to 0.9497 for the weak-signal channels, highlighting its robustness in capturing nonlinear spectral dependencies.

The integration of cGAN-based data augmentation plays a pivotal role in enhancing model reliability, particularly under small-sample conditions. A comparative analysis shows that cGAN augmentation substantially reduces the mean absolute error (MAE) values in the weak transitions and improves the overall prediction uniformity (Table 5). For instance, the MAE for Ω_4_ decreases from 0.2342 to 0.1504, and similar improvements are observed in the oscillator strengths, underscoring the effectiveness of the generated virtual samples in mitigating data sparsity and bias.

**Figure 5:**
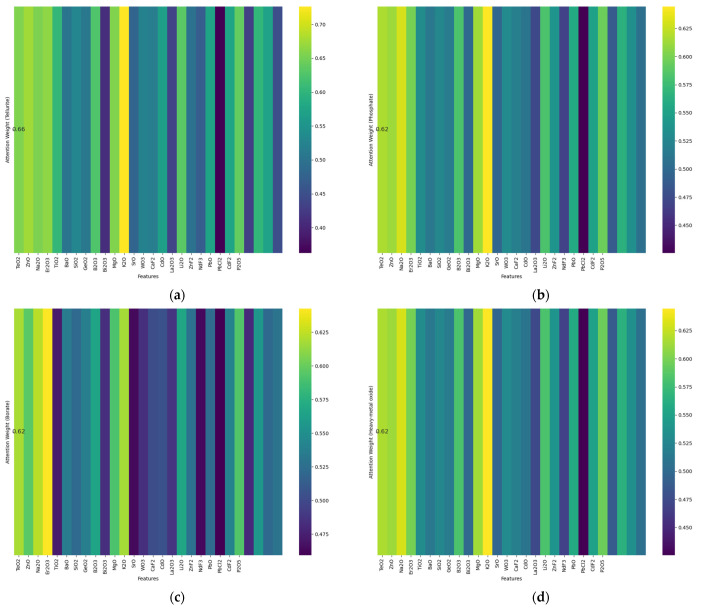
Heatmap of feature attention weights for different glass systems: (**a**) tellurite glass; (**b**) phosphate glass; (**c**) borate glass; (**d**) heavy-metal oxide glass.

Furthermore, the attention weight heatmaps (Figure 5) provide interpretable insights into the model’s decision-making process. In tellurite glasses (Figure 5a), high attention is assigned to network formers like TeO_2_ and modifiers such as ZnO, consistent with their influence on local coordination symmetry. Similar patterns emerge in the phosphate (Figure 5b), borate (Figure 5c), and heavy-metal oxide (Figure 5d) systems, where the attention weights align with established glass structural chemistry principles, such as the role of heavy-metal oxides in enhancing polarizability. This interpretability reinforces the model’s physical relevance and utility for guiding material design.

### 4.4. Universality Validation: Dy^3+^-Doped and Nd^3+^-Doped Glasses

To evaluate the transferability of the proposed framework beyond the primary Er^3+^ training system, the trained hybrid model was applied without any architectural modification or parameter retraining to two independent validation datasets: one consisting of approximately 50 Dy^3+^-doped glass samples and another comprising 87 Nd^3+^-doped glass samples. Both datasets span multiple glass families, including the tellurite, borate, phosphate, silicate/germanate, and heavy-metal oxide systems, thereby providing a stringent test of cross-ion and cross-system generalization.

**Figure 6:**
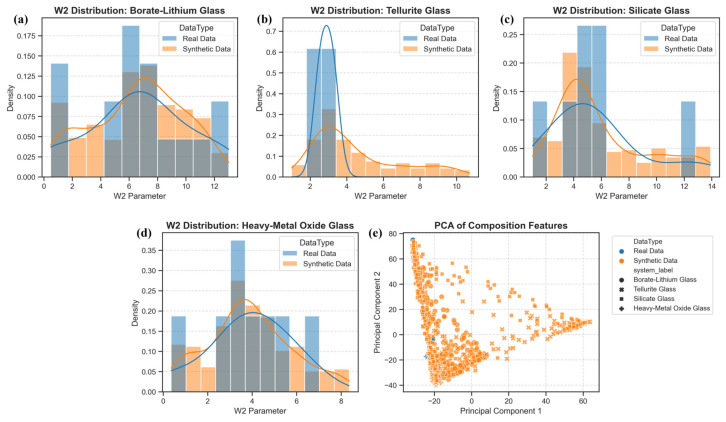
System-resolved Ω_2_ distributions and PCA cluster structure for real and cGAN-generated Dy^3+^-doped glass systems: (**a**) borate-lithium Glass; (**b**) tellurite glass; (**c**) silicate glass; (**d**) heavy-metal oxide glass; (**e**) PCA visualization. (Note: The labels “W2” in the subfigures denote the Judd–Ofelt parameter Ω_2_, with units of 10^−20^ cm^2^).

Figure 6 presents the system-resolved Ω_2_ distributions and PCA cluster structure for the real Dy^3+^-doped glass samples. The marginal distributions (Figure 6a–d) confirm that the Ω_2_ values naturally segregate according to the glass type. The PCA projection (Figure 6e) reveals distinct clustering patterns corresponding to different glass families, demonstrating that the compositional diversity within the Dy^3+^ dataset is well preserved in the feature space learned by the model.

**Figure 7:**
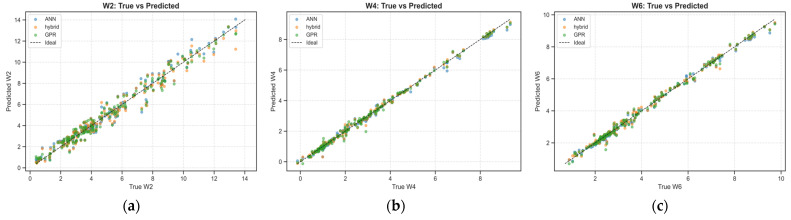
Scatter plots comparing experimental and predicted values for Judd–Ofelt parameters in Dy^3+^-doped glasses: (**a**) Ω_2_; (**b**) Ω_4_; (**c**) Ω_6_. (Note: The notations “W2”, “W4”, and “W6” in the figure titles and axes denote the Judd–Ofelt parameters Ω_2_, Ω_4_, and Ω_6_, respectively. The unit for the original parameters is 10^−20^ cm^2^).

**Figure 8:**
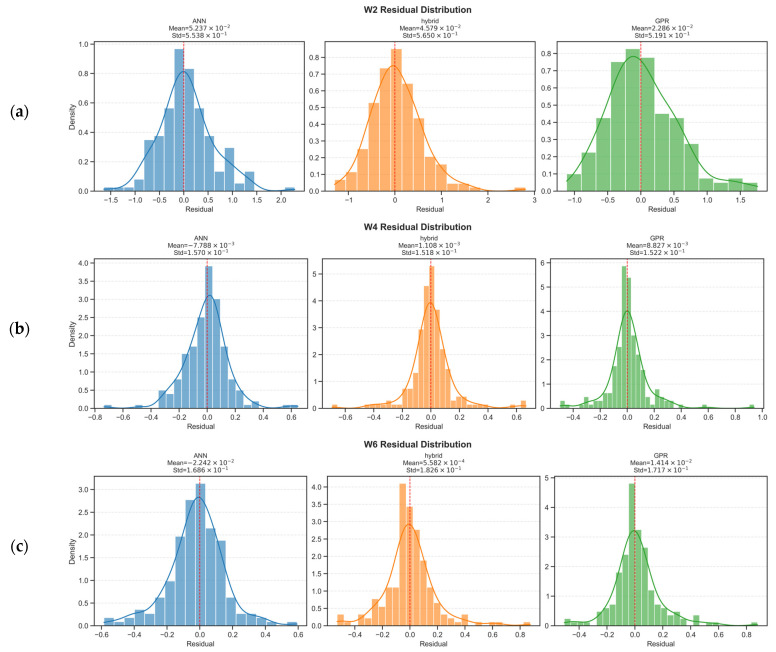
Predicted Residual Distribution of Dy^3+^-Doped Glass: (**a**) Ω_2_; (**b**) Ω_4_; (**c**) Ω_6_. (Note: The notations “W2”, “W4”, and “W6” in the figure titles and axes denote the Judd–Ofelt parameters Ω_2_, Ω_4_, and Ω_6_, respectively. The unit for the original parameters is 10^−20^ cm^2^).

Figure 7 displays scatter plots comparing the experimental and predicted values for the three Judd–Ofelt parameters Ω_2_, Ω_4_, and Ω_6_. The data points lie tightly along the ideal diagonal line, indicating excellent agreement between the prediction and measurement. The corresponding residual distributions (Figure 8) are symmetric, centered near zero, and exhibit narrow standard deviations, confirming the absence of any systematic bias.

Quantitative performance metrics are summarized in Table 6. The hybrid (SVR-on-embedding) model achieves coefficients of determination R^2^ = 0.9668, 0.9961, and 0.9938 for Ω_2_, Ω_4_, and Ω_6_, respectively, with the corresponding mean absolute errors of 0.4198, 0.0887, and 0.1142. Notably, the hybrid approach slightly outperforms both the standalone ANN and a Gaussian Process Regression (GPR) baseline across all three parameters, underscoring the benefit of the two-stage embedding refinement strategy. The exceptionally high R^2^ values for Ω_4_ and Ω_6_ demonstrate that the model captures the subtle compositional dependencies governing these parameters with high fidelity, even on a completely unseen rare-earth ion.

**Figure 9:**
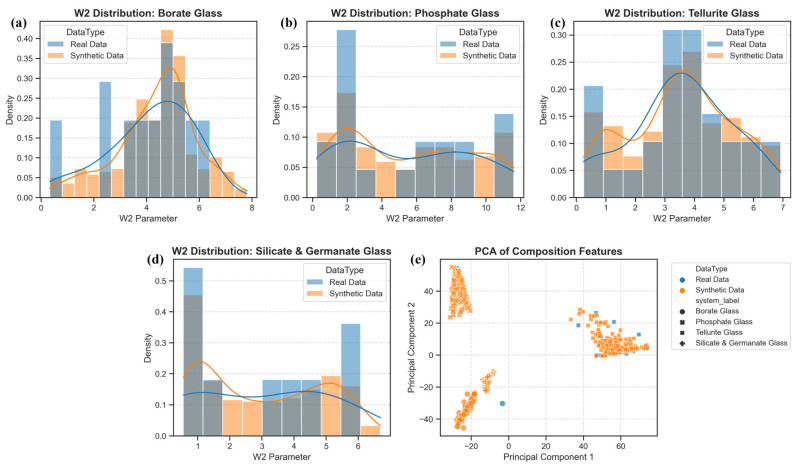
System-resolved Ω_2_ distributions and PCA cluster structure for real and cGAN-generated Nd^3+^-doped glass systems: (**a**) borate glass; (**b**) phosphate glass; (**c**) tellurite glass; (**d**) silicate and germanate glass; (**e**) PCA visualization. (Note: The labels “W2” in the subfigures denote the Judd–Ofelt parameter Ω_2_, with units of 10^−20^ cm^2^).

**Figure 10:**
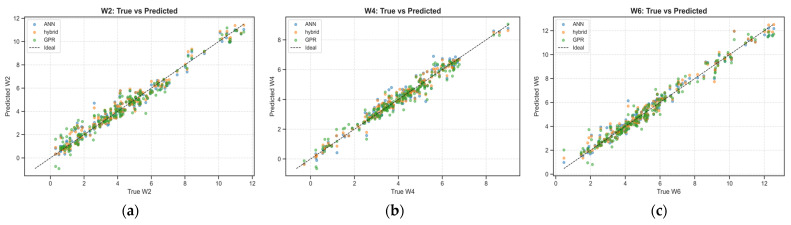
Scatter plots comparing experimental and predicted values for Judd–Ofelt parameters in Nd^3+^-doped glasses: (**a**) Ω_2_; (**b**) Ω_4_; (**c**) Ω_6_. (Note: The notations “W2”, “W4”, and “W6” in the figure titles and axes denote the Judd–Ofelt parameters Ω_2_, Ω_4_, and Ω_6_, respectively. The unit for the original parameters is 10^−20^ cm^2^).

**Figure 11:**
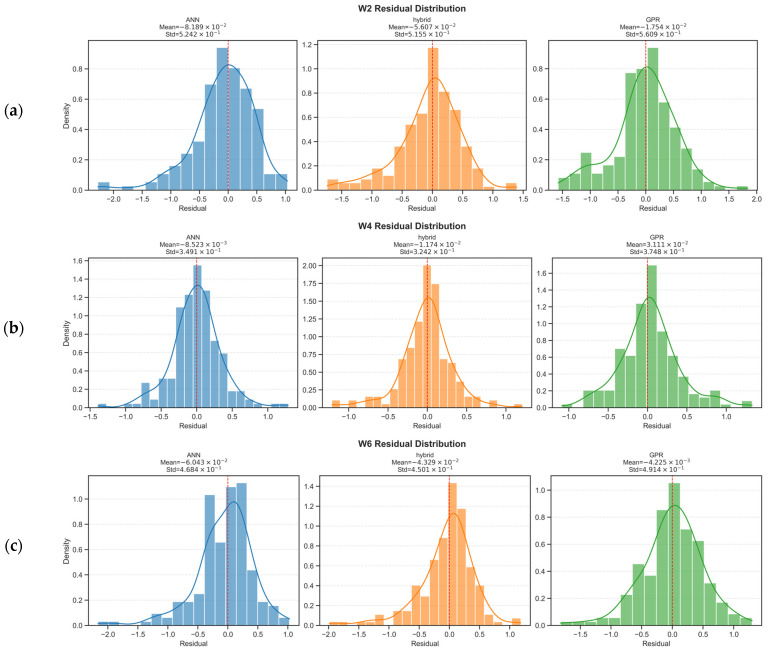
Predicted Residual Distribution of Nd^3+^-Doped Glass. (**a**) Ω_2_. (**b**) Ω_4_. (**c**) Ω_6_. (Note: The notations “W2”, “W4”, and “W6” in the figure titles and axes denote the Judd–Ofelt parameters Ω_2_, Ω_4_, and Ω_6_, respectively. The unit for the original parameters is 10^−20^ cm^2^).

Figure 9, Figure 10 and Figure 11 and Table 7 present analogous results for the Nd^3+^-doped validation dataset. The Ω_2_ distributions and PCA projection (Figure 9) again illustrate clear system-dependent variations, with the phosphate and silicate/germanate glasses occupying distinct regions of the compositional space. The scatter plots (Figure 10) and the residual distributions (Figure 11) reaffirm the model’s predictive robustness: all three Judd–Ofelt parameters are predicted with high accuracy, and residuals remain tightly clustered around zero.

As shown in Table 7, the hybrid model yields R^2^ values of 0.9677, 0.9706, and 0.9741 for Ω_2_, Ω_4_, and Ω_6_, respectively, with the MAE values of 0.3438, 0.2054, and 0.3053. The slightly larger mean absolute error for Ω_6_ in Nd^3+^ compared to Dy^3+^ highlights a known physical distinction among lanthanides. Because Nd^3+^ has a more extended 4f electron cloud than Er^3+^ or Dy^3+^, its Ω_6_ parameter exhibits a stronger, ion-specific sensitivity to host polarizability. Consequently, the Er^3+^-trained compositional embedding cannot perfectly replicate this specific response, leading to a moderate error increase. Nevertheless, the high R^2^ of 0.974 confirms that the model still successfully captures the dominant composition-driven trends, establishing its reliability for cross-ion predictions.

Collectively, these results confirm that the cGAN-augmented attention-hybrid framework, trained exclusively on Er^3+^-doped oxide glasses, transfers effectively to Dy^3+^ and Nd^3+^ systems without task-specific retraining. The high predictive accuracy maintained across three distinct rare-earth ions and five glass families provides strong evidence that the model has learned fundamental composition–property relationships rooted in the underlying physics of the Judd–Ofelt formalism.

### 4.5. Model Superiority and Application Prospects

Specifically, the present work shares partially consistent prediction objectives with previous machine learning studies [8,9,10,11,12], namely the prediction of the Judd–Ofelt parameters Ω_2_, Ω_4_, and Ω_6_. While those studies achieved only moderate accuracy on single glass systems (e.g., Benhadjira et al. reported R^2^ values of 0.79, 0.80, and 0.85 for Ω_2_, Ω_4_, and Ω_6_ using Gaussian Process Regression on Er^3+^-doped oxide glasses) [8], our cGAN-enhanced attention-hybrid regression framework delivers significantly superior performance. For Er^3+^-doped glasses, the hybrid model achieves R^2^ values of 0.962 for Ω_2_, 0.976 for Ω_4_, and 0.931 for Ω_6_. On the independent Nd^3+^-doped validation set, the corresponding R^2^ values reach 0.968, 0.971, and 0.974. These improvements mainly benefit from the cGAN-based data augmentation and the attention mechanism in the hybrid architecture.

The proposed cGAN-augmented attention-hybrid framework substantially outperforms the baselines under small-sample conditions, offering both high accuracy and interpretability. It provides a robust, general tool for data-driven rare-earth glass design.

From an industrial perspective, this predictive capability is highly valuable for the design of technological processes and products. Specifically, the model can accurately predict how modifications in the dopant concentration or the introduction of specific network modifiers (e.g., varying the ratios of ZnO or heavy-metal oxides) directly dictate the spectral emission intensities and radiative lifetimes of the glass. By effectively predicting these specific effects of doping on optical behavior, the model allows glass manufacturers to virtually screen material compositions prior to physical production, significantly reducing the industry’s reliance on trial-and-error melting procedures.

### 4.6. Limitations

Although the proposed framework demonstrates high accuracy and cross-ion generalization, several limitations should be noted. First, the model relies on literature-derived Judd–Ofelt parameters that follow the original authors’ transition selections and fitting procedures. Consequently, the absolute numerical comparability across publications remains inherently limited without a uniform Combinatorial Judd–Ofelt (C-JO) recalculation [17,19]. Therefore, it is crucial to emphasize that the primary objective of this cGAN-augmented attention-hybrid framework is not to pursue sub-10% absolute precision in parameter calculation—which strictly requires standardized recalculation from the raw absorption spectra—but rather to accurately capture and predict the relative composition–property trends across diverse glass systems.

In the field of materials informatics, machine learning models trained on multi-source, aggregated literature datasets have been demonstrably successful when the goal is mapping broad compositional spaces and identifying macro-level heuristic trends [64]. By focusing on these relative trends, the framework successfully normalizes minor band-assignment discrepancies (such as those potentially present in Ref. [24]) to provide a robust, generalized tool. This enables researchers to rapidly narrow down the compositional candidates during the high-throughput design of rare-earth glasses under real-world, small-sample constraints.

Finally, the current implementation is restricted to oxide glass hosts; the extension to fluoride and chalcogenide systems, as well as the prediction of non-radiative rates and mechanical properties, is planned for subsequent studies.

## 5. Conclusions and Outlook

We have developed and validated a cGAN-enhanced attention-hybrid regression framework for the universal prediction of the Judd–Ofelt intensity parameters and oscillator strengths in Er^3+^, Dy^3+^ and Nd^3+^ doped glasses. By combining conditional generative adversarial network data augmentation with an attention-embedded artificial neural network–support vector regression hybrid model, the proposed approach effectively overcomes the longstanding challenge of small-sample regimes in rare-earth glass spectroscopy.

Quantitatively, the framework achieves outstanding predictive performance. For the Er^3+^-doped oxide glasses, the hybrid model delivers coefficients of determination R^2^ > 0.93 for all three Judd–Ofelt parameters (R^2^ = 0.962 for Ω_2_, 0.976 for Ω_4_, and 0.931 for Ω_6_) and maintains high accuracy across the ten selected oscillator strengths. On the independent Dy^3+^ validation dataset, the corresponding R^2^ values exceed 0.96 for Ω_2_, Ω_4_, and Ω_6_, demonstrating an excellent and robust applicability. Although the real experimental dataset comprises only 67 samples, the cGAN-based augmentation strategy generates approximately 1267 physically consistent virtual samples per system while enforcing non-negativity, renormalization to 100 mol%, Ω_2_ clipping, and teacher-network consistency. This augmentation demonstrably enhances the generalization, reducing the mean absolute errors by 20–35% for the weak transitions on the independent test set (Table 5) and enabling reliable predictions across four distinct glass systems.

Qualitatively, the attention mechanism embedded in the model provides physically interpretable insights. The feature-weight heatmaps reveal that network formers (e.g., TeO_2_) and modifiers (e.g., ZnO, BaO) dominate the prediction of Ω_2_, consistent with their influence on the local coordination symmetry, while the polarizable heavy-metal oxides exert the strongest effect on Ω_6_ through the host polarizability. These attention patterns align closely with the established glass chemistry principles, confirming that the data-driven model captures genuine structure–property relationships rather than spurious correlations.

A fundamental limitation of this and similar literature-based approaches is the potential inconsistency in the absolute Judd–Ofelt parameter values arising from varying fitting methodologies, transition choices, and band assignments across the original publications. Such discrepancies may introduce errors and represent a bottleneck for high-precision applications. Nevertheless, the strong performance and generalization achieved here demonstrate the reliable learning of physically meaningful trends.

The accurate, interpretable, and generalizable predictions obtained in this work will accelerate the rational design of rare-earth-doped glasses for high-performance optical fiber sensors, fluorescence-intensity-ratio temperature sensors, and optical bio-probes. Future extensions will include the fluoride and chalcogenide hosts, the incorporation of non-radiative decay rates and mechanical properties, as well as the inverse-design capabilities via generative models. The open-source LOMS.cz platform will also be employed for a standardized combinatorial Judd–Ofelt recalculation, further enhancing dataset consistency and reproducibility. Overall, the present framework offers a powerful, physics-informed tool that bridges small-sample constraints with high-throughput materials discovery, paving the way for next-generation rare-earth photonic devices.

## Figures and Tables

**Table 1:** Composition of Selected Glass Samples (mol %).

Composition	Samples				
	S1	S2	S3	S4	S5
TeO_2_	60	60	60	60	60
ZnO	5	5	5	5	5
Na_2_O	0	0	0	0	0
Er_2_O_3_	1	2.5	3.5	4	5
TiO_2_	0	0	0	0	0
BaO	34	32.5	31.5	31	30
SiO_2_	0	0	0	0	0
GeO_2_	0	0	0	0	0
B_2_O_3_	0	0	0	0	0
Bi_2_O_3_	0	0	0	0	0
MgO	0	0	0	0	0
K_2_O	0	0	0	0	0
SrO	0	0	0	0	0
WO_3_	0	0	0	0	0
CaF_2_	0	0	0	0	0
CdO	0	0	0	0	0
La_2_O_3_	0	0	0	0	0
Li_2_O	0	0	0	0	0
ZnF_2_	0	0	0	0	0
NdF3	0	0	0	0	0
PbO	0	0	0	0	0
PbCl_2_	0	0	0	0	0
CdF_2_	0	0	0	0	0
P_2_O_5_	0	0	0	0	0

**Table 2:** Experimental (*f*_exp_) and calculated (*f*_cal_) oscillator strengths (×10^−6^) for transitions from the ground state ^4^I_15_/_2_, and J–O parameters of selected samples.

Parameters	Samples				
	S1	S2	S3	S4	S5
*f*_exp_(^4^I_15/2_ → ^4^I_13/2_)	3.9908	3.7456	3.6813	3.247	2.8191
*f*_cal_(^4^I_15/2_ → ^4^I_13/2_)	3.3459	3.1693	3.1089	2.7539	2.3945
*f*_exp_(^4^I_15/2_ → ^4^I_11/2_)	0.3231	0.3006	0.2503	0.2577	0.2317
*f*_cal_(^4^I_15/2_ → ^4^I_11/2_)	1.0253	0.961	0.939	0.8296	0.7093
*f*_exp_(^4^I_15/2_ → ^4^F_9/2_)	0.7526	0.6995	0.6854	0.6028	0.6628
*f*_cal_(^4^I_15/2_ → ^4^F_9/2_)	0.6191	0.5833	0.5755	0.5004	0.5665
*f*_exp_(^4^I_15/2_ → ^2^H_11/2_)	2.1871	1.7294	1.5859	1.3064	1.1211
*f*_cal_(^4^I_15/2_ → ^2^H_11/2_)	2.1401	1.6915	1.5499	1.2786	1.1024
*f*_exp_(^4^I_15/2_ → ^4^F_7/2_)	0.22	0.2949	0.3038	0.2725	0.2552
*f*_cal_(^4^I_15/2_ → ^4^F_7/2_)	1.3524	1.2839	1.2612	1.1162	0.997
Ω_2_	3.2083	2.5738	2.3684	1.977	1.5568
Ω_4_	0.8844	0.8497	0.8289	0.7546	0.3942
Ω_6_	2.3659	2.2483	2.2073	1.9584	1.6836

**Table 3:** Kolmogorov–Smirnov statistics comparing real and cGAN-generated distributions of key glass components and Ω_2_.

Variable	KS Statistic	*p*-Value
TeO_2_	0.43	1 × 10^−3^
ZnO	0.32	3 × 10^−3^
Na_2_O	0.39	7 × 10^−4^
Er_2_O_3_	0.32	5 × 10^−3^
Ω_2_	0.16	7 × 10^−2^

**Table 4:** $R^{2}$ and MAE of each parameter for Er-doped glasses.

Parameter	R^2^ (ANN)	MAE (ANN)	R^2^ (Hybrid)	MAE (Hybrid)
Ω_2_	0.956602	0.727906	0.962373	0.489089
Ω_4_	0.941645	0.18698	0.975633	0.150411
Ω_6_	0.905107	0.13899	0.930871	0.120452
*f*_exp_(^4^I_15/2_ → ^4^I_13/2_)	0.819532	0.493275	0.832229	0.413286
*f*_cal_(^4^I_15/2_ → ^4^I_13/2_)	0.787721	0.506865	0.803392	0.381233
*f*_exp_(^4^I_15/2_ → ^4^I_11/2_)	0.929496	0.273869	0.949732	0.225317
*f*_cal_(^4^I_15/2_ → ^4^I_11/2_)	0.863342	0.447153	0.89189	0.400413
*f*_exp_(^4^I_15/2_ → ^4^F_9/2_)	0.943191	0.295321	0.945729	0.291678
*f*_cal_(^4^I_15/2_ → ^4^F_9/2_)	0.941327	0.28558	0.947863	0.274817
*f*_exp_(^4^I_15/2_ → ^2^H_11/2_)	0.773030	1.565148	0.803388	1.473475
*f*_cal_(^4^I_15/2_ → ^2^H_11/2_)	0.779249	1.457181	0.808932	1.375028
*f*_exp_(^4^I_15/2_ → ^4^F_7/2_)	0.748836	0.519048	0.792704	0.467144
*f*_cal_(^4^I_15/2_ → ^4^F_7/2_)	0.729253	0.503337	0.761065	0.454801

**Table 5:** Comparison of ANN performance with and without cGAN data augmentation on the strictly independent test set.

Parameter	R^2^ (No-cGAN)	MAE (No-cGAN)	R^2^ (cGAN)	MAE (cGAN)
Ω_2_	0.967762	0.439491	0.962373	0.489089
Ω_4_	0.949713	0.234241	0.975633	0.150411
Ω_6_	0.898537	0.124964	0.930871	0.120452
*f*_exp_(^4^I_15/2_ → ^4^I_13/2_)	0.765574	0.492909	0.832229	0.413286
*f*_cal_(^4^I_15/2_ → ^4^I_13/2_)	0.792802	0.394512	0.803392	0.381233
*f*_exp_(^4^I_15/2_ → ^4^I_11/2_)	0.904054	0.309634	0.949732	0.225317
*f*_cal_(^4^I_15/2_ → ^4^I_11/2_)	0.80397	0.447159	0.89189	0.400413
*f*_exp_(^4^I_15/2_ → ^4^F_9/2_)	0.929002	0.30847	0.945729	0.291678
*f*_cal_(^4^I_15/2_ → ^4^F_9/2_)	0.941989	0.282145	0.947863	0.274817
*f*_exp_(^4^I_15/2_ → ^2^H_11/2_)	0.783527	1.553795	0.803388	1.473475
*f*_cal_(^4^I_15/2_ → ^2^H_11/2_)	0.820255	1.337511	0.808932	1.375028
*f*_exp_(^4^I_15/2_ → ^4^F_7/2_)	0.765729	0.498262	0.792704	0.467144
*f*_cal_(^4^I_15/2_ → ^4^F_7/2_)	0.746838	0.486488	0.761065	0.454801

**Table 6:** $R^{2}$ and MAE of each parameter for Dy^3+^-doped glasses.

Parameter	R^2^ (ANN)	MAE (ANN)	R^2^ (Hybrid)	MAE (Hybrid)	R^2^ (GPR)	MAE (GPR)
Ω_2_	0.9666	0.4319	0.9668	0.4198	0.9718	0.4042
Ω_4_	0.9953	0.0933	0.9961	0.0887	0.9952	0.0935
Ω_6_	0.9937	0.1175	0.9938	0.1142	0.9937	0.1146

**Table 7:** $R^{2}$ and MAE of each parameter for Nd^3+^-doped glasses.

Parameter	R^2^ (ANN)	MAE (ANN)	R^2^ (Hybrid)	MAE (Hybrid)	R^2^ (GPR)	MAE (GPR)
Ω_2_	0.9663	0.3574	0.9677	0.3438	0.9534	0.4197
Ω_4_	0.9549	0.2546	0.9706	0.2054	0.9494	0.2703
Ω_6_	0.9729	0.3221	0.9741	0.3053	0.9650	0.3708

## Data Availability

Data are contained within the article.

## Abbreviations

The following abbreviations are used in this manuscript:

cGAN	Conditional Generative Adversarial Network
